# Accidental oxygen disconnection in the emergency department

**DOI:** 10.4103/0974-2700.62123

**Published:** 2010

**Authors:** Guyon J Hill, Bruce D Adams

**Affiliations:** Department of Emergency Medicine, Brooke Army Medical Center, El Paso, TX, USA; 1 William Beaumont Army Medical Center, El Paso, TX, USA

**Keywords:** Airway management, bag-valve mask, breathing, hypoxia, intubation, oxygen, safety, training

## Abstract

Accidental oxygen disconnection during rapid sequence intubation (RSI) in the emergency department is a potentially catastrophic yet avoidable event. We report three cases of inadvertent oxygen disconnection during RSI, which resulted in significant oxygen desaturation. This error can potentially be prevented by thorough preparation, focusing on teamwork training, ensuring an ergonomic environment, and by making simple modifications to existing equipment.

## INTRODUCTION

Rapid sequence intubation (RSI) is necessary for resuscitating critically ill patients in the emergency department (ED). The process of emergency intubation, however, is fraught with the potential for life-threatening errors that include equipment failure, medication errors, procedural complications, and teamwork mishaps.[1–3]

The specific complication of oxygen desaturation is the most common complication during RSI and it has multiple causes.[4] While accidental disconnection of the oxygen circuit has been reported for stabilized patients on ventilators in both intensive care units and operating rooms, this complication has not previously been reported during RSI or in the ED. Historically, the majority of adverse outcomes relating to these types of errors have been death or permanent brain damage. In one study investigating the reasons for these adverse outcomes, misconnection or disconnection of the breathing circuit was the greatest reason for adverse outcomes (35%).[5] Other studies have shown that disconnection of the breathing circuit was the most common form of human error resulting in avoidable[6,7] One of these studies also concluded that up to a BVM while making adjustments to the ventilator. Severalone-third of disconnections were caused by interference in the breathing circuit by a third party. We present a series of accidental oxygen desaturation events caused by inadvertent oxygen circuit disconnections in the ED.

## CASE REPORTS

### Case 1

A 46-year-old female arrived by ambulance for acute respiratory failure with a respiratory rate of 40-50 respirations per minute. The medical emergency team prepared for RSI by preoxygenating the patient with a nonrebreather mask at 100% FiO_2_. The first attempt at intubation was unsuccessful, but the respiratory therapist had also switched the oxygen source to the ventilator without the physician's knowledge. The oxygen saturation declined to around 70% during several attempts at RSI, despite the use of a rescue bag-valve mask (BVM) and a nasopharyngeal airway. The physician then discovered that the BVM was attached to the air outlet instead of the oxygen outlet. The physician reconnected the patient's BVM to the oxygen supply and the patient's oxygen saturation recovered to 100%. No permanent injury was noted as a result of the hypoxic event.

### Case 2

A 13-month-old male was intubated for respiratory distress secondary to pneumonia. Following successful RSI and placement on a ventilator, the ED physicians briefly left the bedside. The respiratory therapist ventilated the patient using a BVM while making adjustments to the ventilator. Several minutes later, the oxygen saturation plummeted to approximately 30% with a pulse rate of 30-40 beats per minute. The medical emergency team leader quickly discovered that the BVM had been inadvertently disconnected from high-flow oxygen. Upon reconnection to supplemental oxygen, the patient's oxygen saturation and heart rate rapidly returned to normal and the patient suffered no adverse outcome.

### Case 3

A 55-year-old patient was brought to the ED after suffering burns and smoke inhalation following a house fire. Despite accurate confirmation of endotracheal tube placement with end tidal CO_2_ and auscultation, the patient rapidly desaturated to 83%. It was noted at that time that the BVM had been disconnected from oxygen during the RSI process. The BVM was connected to oxygen and the patient's oxygen saturation improved immediately. The patient suffered no ill effects from the period of hypoxia.

## DISCUSSION

The cases presented here were caused by either a disconnection of supplemental oxygen or attaching a device to the wrong connection during the RSI process. Teamwork and provider errors far exceed equipment failure as contributors to preventable patient morbidity and mortality.[1–3] Indeed, more than half of the death and disability that occurs in the ED may be a result of team errors.[1,2,8] Airway management is particularly susceptible to team errors.[4,9] One retrospective analysis to investigate the role of human error in airway management showed that 82% of adverse patient outcomes were from human error, while equipment failure was only responsible for 14% of incidents.[6]

Standardizing tasks and clear communication can reduce error. For example, to prevent the communication error seen in Case 1, one should assign the task of switching the solitary oxygen source from the nonrebreather mask to the ventilator once the endotracheal tube is in place. Training in airway skills usually focuses on individuals, although real-time airway management is seldom, if ever performed in isolation. There may also be students of varying types present as well as residents of differing skill levels and experience. Minimizing the interference of third parties not directly involved with the patient can also reduce the possibility of interference with the oxygen source.

Ergonomic improvements in airway equipment may also lessen the likelihood of desaturation. Many hospitals have only one oxygen source or possibly less for each bed. As seen in these cases, this setup can result in accidental desaturation. Ensuring one outlet for each device that supplies oxygen during RSI will minimize the risk of disconnection. These connections should be as secure as possible or permanent. All oxygen outlets should remain visible to the resuscitation team leader and should not be obscured during RSI by people or equipment. Direct visualization of the oxygen circuit could have prevented the desaturation that occurred in all cases. Vigilance must be maintained during RSI due to the addition of care providers and materials. In some instances, the installation of dual oxygen flow devices may be beneficial. These allow for multiple airway devices to be attached to a single wall source. This allows for the nonrebreather mask and the BVM to be attached simultaneously during the preparation stage. This will minimize the opportunity of not connecting (or double switching) the devices during the procedure.

## CONCLUSION

Many preventable errors that occur during a patient's hospital stay may occur during airway management. Improvements in the functioning of the airway team can improve patient safety and decrease the likelihood of significant adverse events. Changes in the environment in which airway management takes place may also help to decrease accidental oxygen desaturation. Direct visualization of the entire airway circuit, adequate and secure outlets and attachments, and oxygen flow splitting devices can all be beneficial.
